# First Genome Sequence of *Leptospira interrogans* Serovar Pomona, Isolated from a Bovine Abortion

**DOI:** 10.1128/genomeA.00345-16

**Published:** 2016-05-19

**Authors:** Vanina Varni, Ariel Koval, Ariel Nagel, Paula Ruybal, Karina Caimi, Ariel F. Amadio

**Affiliations:** a Instituto de Biotecnología, Instituto Nacional de Tecnología Agropecuaria, Hurlingham, Buenos Aires, Argentina; b Consejo Nacional de Investigaciones Científicas y Técnicas (CONICET), Buenos Aires, Argentina; c Biogenesis Bagó, Garín, Buenos Aires, Argentina; d Instituto de Microbiología y Parasitología Médica, Facultad de Medicina, Universidad de Buenos Aires/CONICET, Buenos Aires, Argentina; e Estación Experimental Agropecuaria Rafaela, Instituto Nacional de Tecnología Agropecuaria, Rafaela, Santa Fe, Argentina

## Abstract

Leptospirosis is a widespread zoonosis and a re-emergent disease of global distribution with major relevance in veterinary production. Here, we report the whole-genome sequence of *Leptospira interrogans* serovar Pomona strain AKRFB, isolated from a bovine abortion during a leptospirosis outbreak in Argentina.

## GENOME ANNOUNCEMENT

Leptospirosis is caused by spirochetes of the genus *Leptospira* (1). Serological classification indicates the presence of over 200 pathogenic serovars (2). The manifestations in livestock are mainly reproductive problems such as infertility and abortion (3). Cattle are usually maintenance hosts of serovar Hardjo throughout the world (4, 5), but the presence of other serovars was also demonstrated (6, 7). Remarkably, in Argentina the most frequent serovar in bovines is Pomona (8–11), while in other countries it is mostly associated with swine (12). However, there is scarce information about host-pathogen interactions with this serovar during livestock leptospirosis outbreaks. Thus, the availability of new genomic *Leptospira* sequences obtained from bovine isolates provides additional data for better understanding of this pathogen.

This work reports the draft genome sequence of *Leptospira interrogans* serovar Pomona strain AKRFB. The strain was isolated from a fetal bovine kidney in 2007, during a leptospirosis outbreak that affected a dairy herd in Buenos Aires, Argentina. The strain was characterized by serological and molecular methods. It belonged to serogroup Pomona and its associated genotype in Argentina (ST52) (10, 11). When evaluated in an animal model, the strain presented high virulence and caused neurological symptoms (13). Therefore, the strain was further evaluated as a candidate vaccine, satisfactorily protecting animals against challenge with the commercial vaccine and the other 3 field isolates (14). Upon incorporation of AKRFB to new commercial vaccines, there have been no records of isolates belonging to serogroup Pomona in vaccinated herds.

Genomic DNA was isolated using a standard chloroform isoamyl-alcohol extraction. Paired-end Nextera XT libraries were constructed and sequenced in an Illumina MiSeq sequencer. The quality trimming (15) applied to raw reads yielded 2,664,724 paired sequences. *De novo* assembly was done using SPAdes v3.6.2 (16) and reported 102 contigs >500 bp, the largest being 352,992 bp, with an *N*_50_ of 91,263 bp. Scaffolds were oriented using ABACAS (17) with the genome of *Leptospira interrogans* serovar Lai strain 56601 (18) as a reference. The final assembly comprised ~4.63 Mbp, 3,763 predicted genes, 37 tRNA copies, and a G+C content of 34.56%. The chromosome II was assembled into a single contig by SPAdes. The genome was annotated using PROKKA (19). The annotated scaffolds were compared with the same reference using BLAST and ACT (20) to analyze structural and gene content differences.

An MLST analysis (http://pubmlst.org/leptospira/) associated AKRFB with ST52. This profile also showed correlation to serogroup Pomona in worldwide strains as described previously (10).

ISFinder (21) was used to predict insertion sequences in the AKRFB draft genome as IS1500B, ISLin2, IS1500A, and, IS1501. The genome presents previously described virulence gene candidates, such as *lip*L32, *lip*L41, *lig*A, and *lig*B (2).

To our knowledge this is the first report of a complete sequence belonging to serogroup Pomona from a bovine abortion strain and the first sequence from an Argentinian isolate.

### Nucleotide sequence accession numbers.

This whole-genome shotgun project has been deposited at DDBJ/ENA/GenBank under the accession number LUHH00000000. The version described in this paper is version LUHH01000000.
